# Evidence of Microvascular Changes in the Retina following Kawasaki Disease

**DOI:** 10.1038/srep40513

**Published:** 2017-01-17

**Authors:** Katherine Y. H. Chen, David P. Burgner, Tien Y. Wong, Seang Mei Saw, Swee Chye Quek, Audrey Y. C. Pang, Seo Wei Leo, Inez B. Wong, Diana Zannino, Nigel Curtis, Michael Cheung, Carol Y. Cheung, Terence C. W. Lim

**Affiliations:** 1 Murdoch Childrens Research Institute, The Royal Children’s Hospital, Melbourne, Vic, Australia; 2 Department of Paediatrics, University of Melbourne, Vic, Australia; 3 Infectious Diseases Unit and Department of General Medicine, The Royal Children’s Hospital Melbourne, Vic, Australia; 4 Department of Paediatrics, Monash University, Vic, Australia; 5 Singapore Eye Research Institute, Singapore National Eye Centre, Singapore; 6 Duke-NUS Medical School, National University of Singapore, Singapore; 7 Department of Paediatrics, Yong Loo Lin School of Medicine, National University of Singapore, Singapore; 8 Khoo Teck Puat-National University Children’s Medical Institute, National University Health System, Singapore; 9 Department of Ophthalmology, Tan Tock Seng Hospital, Singapore; 10 Dr Leo Adult &Paediatric Eye Specialist, Mount Elizabeth Hospital, Singapore; 11 Department of Ophthalmology, National University Health System, Singapore; 12 Heart Research Group, Murdoch Childrens Research Institute and Department of Cardiology, The Royal Children’s Hospital, Melbourne, Vic, Australia; 13 Department of Ophthalmology and Visual Sciences, The Chinese University of Hong Kong, Hong Kong

## Abstract

It is unclear whether all children with Kawasaki disease (KD) have increased later cardiovascular risk. The retinal microvasculature reflects changes in the microcirculation and is associated with traditional cardiovascular risk factors and events. The aim of this study was to investigate retinal microvascular parameters in two populations of patients with previous KD and control participants. We performed case-control studies of 116 (57 patients and 59 control participants) Australian and 156 (78 patients and 78 control participants) Singaporean individuals, at least two years since their acute illness. Standardised retinal photographs were graded by trained technicians using a semi-automated software, which quantifies the retinal microvasculature (calibre, branching angle, fractal dimensions, and tortuosity). Retinal venules of Singaporean KD patients were 9.67 μm (95% CI 4.87 to 14.51, p < 0.001) larger than control participants following correction for traditional cardiovascular risk factors. An incremental increase in the size of retinal venules in those with coronary artery abnormalities was observed. There was limited evidence that retinal venules were larger in Australian KD patients with coronary artery abnormalities compared to control participants (7.34 μm, 95% CI 1.30 to 15.99, p = 0.10). Differences in retinal microvasculature were particularly evident in Singaporean KD patients. Larger retinal venules may reflect chronic inflammation and endothelial dysfunction, and are associated with coronary artery disease in adults.

Kawasaki disease (KD) is an acute systemic paediatric vasculitis of unknown aetiology first described in 1967^1^. The incidence of KD is increasing and it is the commonest cause of acquired heart disease of children in industrialised countries^2^. Whether KD causes changes to the systemic vasculature indicative of later cardiovascular risk, especially in those without identified coronary artery changes or with regressed coronary artery lesions, is unknown. Studies investigating this question have used non-invasive assessment of large artery structure and function extrapolated from studies of subclinical atherosclerosis. Findings from studies of carotid intima-media thickness, pulse wave velocity, and endothelial function of peripheral arteries following KD are conflicting, particularly in the low risk groups^3^.

KD is considered a medium sized vasculitis causing coronary artery aneurysms in the most severely affected cases. However, vascular involvement may be systemic and aneurysms occasionally occur in the aorta, axillary, brachial and iliac arteries^4^. Whether the microcirculation is affected following KD has not been reported previously.

Changes to the microcirculation can be quantified by non-invasive assessment of the retinal microvasculature, a technique increasingly used to augment cardiovascular risk assessment in clinical and research studies in both adults and children^5^,^6^. The calibre of the retinal vessels is the most widely studied retinal microvascular parameter with good reproducibility^7^,^8^. Smaller retinal arterioles are associated with traditional cardiovascular risk factors^9^, and incident cardiovascular disease^10^. Larger retinal venules are associated with markers of inflammation^11^,^12^,^13^, traditional cardiovascular risk factors^9^,^14^, endothelial dysfunction^9^, and increased risk of coronary heart disease^15^ and stroke in adults^16^. Other retinal microvascular parameters, such as retinal fractal dimension and tortuosity, have also been associated with cardiovascular disease^16^,^17^,^18^. This accumulating evidence suggests that retinal microvascular parameters may be predictive of cardiovascular risk. Associations between retinal microvascular parameters and cardiovascular risk factors have also been described in children^5^,^14^, and limited longitudinal data have linked childhood adverse cardiovascular risk factors with changes in the retinal microvascular parameters in adulthood^19^.

The aim of this study was to investigate retinal microvascular parameters (calibre, branching angle, fractal dimensions, and tortuosity) in two populations (predominantly Caucasian and Asian) of patients with previous KD and control participants. We hypothesised that KD patients would have an abnormal retinal microvascular profile (e.g. larger venules, smaller arterioles, suboptimal fractal dimension and tortuosity) compared to control participants.

## Methods

We designed and performed two case-control studies of children with previous KD and control participants in Australia and Singapore. The studies were performed in accordance with the Declaration of Helsinki and relevant local guidelines and regulations. Written consent was obtained from children’s guardians or adult participants prior to enrolment. In both cohorts, ethnicity was based on self-report of the participants’ biological parents.

### Australian cohort

Patients aged six to 30 years who had acute KD at least two years prior were recruited from The Royal Children’s Hospital and Monash Medical Centre, Melbourne, Australia. The study was approved by the Human Research Ethics Committee of both hospitals. Individuals with a history of acute KD that fulfilled either the American Heart Association diagnostic criteria for KD^20^, and/or had abnormal coronary artery dimensions that met the Japanese Ministry of Health Criteria^21^ on an echocardiogram done within two months of disease onset were included in the KD group. Kawasaki disease patients were further categorised into those with always normal coronary arteries and those with coronary artery abnormalities based on their worst ever echocardiogram.

Control participants were unrelated healthy individuals of similar age and sex to KD patients recruited from the outpatient department of The Royal Children’s Hospital Melbourne, children of staff members, or unrelated friends of KD patients. Exclusion criteria in both groups were pregnancy, diabetes, known atherosclerotic cardiovascular disease, treatment for hypertension and/or hyperlipidaemia, or chronic inflammatory conditions.

After a minimum six-hour fast, participants attended Murdoch Childrens Research Institute for a single appointment between September 2013 and December 2015, during which demographic details, anthropometric and adiposity measurements (BC 418, Tanita, Tokyo, Japan), and blood pressure (SphygmoCor^®^ XCEL, AtCor Medical Pty Ltd, NSW, Australia) were obtained. Retinal photographs were taken using a non-mydriatic retinal camera (CR6-45NM, Canon, Tokyo, Japan or model VX 10i KOWA, Tokyo, Japan). Disc centred photographs of the retina were taken of the left and right eyes without mydriasis. High sensitivity C-reactive protein (hsCRP) on blood obtained at the study visit was measured using standardised procedures (Abbott Architect, IL, USA).

### Singaporean cohort

Kawasaki disease patients aged six to 18 years were recruited from the National University Hospital, Singapore. The case definition for Kawasaki disease and coronary artery abnormality used were identical to the Australian cohort. The study was approved by the hospital research ethics board. Demographic details, height, weight (Seca, Hamburg, Germany), and blood pressure (GE Procare 400, USA) were recorded. High-resolution digital retinal photographs centered on the disc and macula were taken using standardised settings (6.3 megapixel, resolution 3072 × 2048, CR6 Canon Japan) between June 2010 and July 2013.

Control participants of similar age and sex were selected from the Singaporean Cohort Study of Risk Factors for Myopia. Details of the study population and recruitment procedures are described in detail elsewhere^22^. Briefly, schoolchildren from three schools in Singapore aged seven to nine years at baseline were enrolled and examined in 2001 and 2006. Children with cardiovascular disease, malignancy, syndrome-associated myopia, or underlying eye disorders were excluded. The Ethics Committee of the Singaporean Eye Research Institute approved of the study. After pupil dilatation with cyclopentolate 1%, disc centered retinal photographs were taken using a Canon CR6-45NM non mydriatic camera (Tochigiken, Japan). High sensitivity C-reactive protein was not measured in the Singaporean cohort.

### Measurement of retinal microvascular parameters

**Figure 1:**
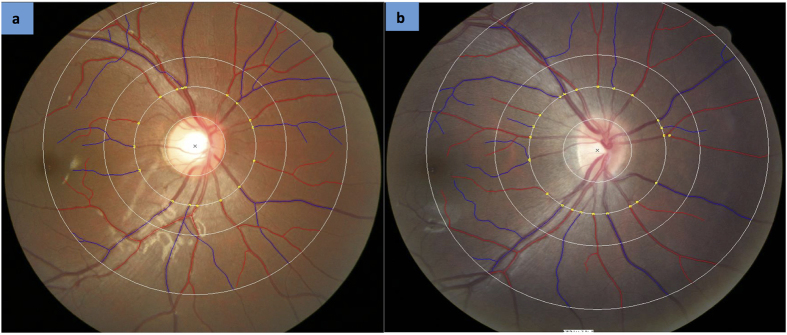
SIVA image. Retinal photograph of a Singaporean (**a**) and Australian (**b**) participant measured using Singapore I Vessel Assessment (SIVA). Arterioles are highlighted in *red* and venules in *blue*. The measured area of retinal microvascular parameters is demarcated by white circles 0.5 to 2.0 disc diameters away from the disc margin.

All retinal photographs from both cohorts were analysed at the Singapore Eye Research Institute using a semi-automated computer-assisted program (Singapore I Vessel Assessment [SIVA], version 3.0, School of Computing, National University of Singapore)^23^. The following retinal microvascular parameters were quantified by trained graders, blinded to the participant’s grouping using a standardised protocol: retinal microvascular calibre, branching angle, fractal dimension, and tortuosity of both arterioles and venules. The measured area was defined as the region from 0.5 to 2.0 disc diameters away from the disc margin. All visible vessels coursing through the specified zone were measured (Fig. 1). Reliability of the quantitative measurements of retinal microvasculature using the SIVA program has been reported before^24^. Table 1 summarizes the method of measurement for each parameter.

### Statistical methods

Statistical analysis was done using Stata 13.0 (Stata Corporation, College Station, TX, USA). Measures of the retinal microvascular parameters and hsCRP were analysed as continuous variables. Due to the skewed distribution of hsCRP, arteriolar and venular tortuosity, these variables were log-transformed. Univariate comparisons of continuous variables were made using independent sample 2-tailed *t*-test. Multi-variable linear regression was used to quantify the differences between groups adjusting for age, sex, mean arterial blood pressure, and body mass index. Sensitivity analysis of participants with more homogenous ethnicity (e.g. Chinese only in the Singaporean cohort and European-Caucasian only in the Australian cohort) was done to investigate the effect of ethnicity. Finally, the microvascular calibre was added to the regression model for tortuosity to investigate if the reduction in vessel tortuosity is due to larger vessel calibre. This study was reported following the STROBE guidelines for reporting observational studies.

## Results

One hundred and twenty participants (60 KD and 60 control participants) were recruited in Australia. One KD patient had an ungradable retinal photograph due to poor image quality and families of two KD patients and one control participant did not consent to have their retinal photograph sent overseas for grading. There were 78 KD patients recruited in Singapore and 78 control participants selected from the Singaporean Cohort Study of Risk Factors for Myopia; all images were gradable.

The Singaporean control participants were marginally older compared to KD patients and had higher systolic and diastolic blood pressures (Table 2). The Australian cohort was predominantly European-Caucasian. There were no differences between the Singaporean cohort in the distribution of Chinese, Malay and Indian participants.

Compared to the Singaporean cohort, the Australian cohort included over three times more patients with a history of coronary artery abnormalities, including 8 (14.0%) with persistent coronary artery aneurysms more than eight mm in diameter (giant aneurysms) (Table 3). There were no participants from Singapore with giant coronary artery aneurysms. The interval between the KD illness to retinal assessment was longer in the Australian KD patients compared to the Singaporean ones (11.8 ± 5.9 versus 6.9 ± 4.0 years, p < 0.001). There were no differences in hsCRP levels in Australian participants with previous KD compared to control participants, although a trend towards higher hsCRP in those with coronary artery abnormality was observed (Table 2).

In addition to the above clinical differences, the Australian control group had larger retinal arterioles, smaller fractal dimensions and lesser tortuosity compared to the Singaporean control group (Supplementary 1). Therefore, data from Australia and Singapore were not combined and the results of retinal vascular parameters are therefore presented separately for each cohort.

Kawasaki disease patients from Singapore had larger retinal venules compared to control participants (216.25 ± 14.41 versus 205.31 ± 13.65 μm, p < 0.001), with an incremental increase in those patients with coronary artery abnormalities compared to those with ‘always normal’ coronary arteries (225.63 ± 15.56 versus 215.01 ± 13.60 μm, p = 0.02) (Table 4). There was limited evidence of a similar association in Australian KD patients with coronary artery abnormalities compared to control participants (221.20 ± 15.30 versus 214.38 ± 22.69 μm, p = 0.13). Exploring the association of retinal venular calibre with age in both cohorts, there was limited evidence of a small increase with each year increase in age (0.37 μm, 95% CI 0.04 to 0.79, p = 0.08) (Supplementary 2). There was no association found between the size of the retinal venules and time since KD (average increase of 0.27 μm per year after acute KD, 95% CI 0.19 to 0.74, p = 0.25) (Supplementary 2).

Singaporean KD patients also had larger retinal arterioles (149.43 ± 13.72 versus 140.15 ± 9.75 μm, p < 0.001) and less tortuous retinal arterioles (7.34, 95% CI 7.05 to 7.64 versus 8.70, 95% CI 8.36 to 9.14, p < 0.001) and venules (7.34, 95% CI 7.12 to 7.64 versus 9.42, 95% CI 9.14 to 9.71, p < 0.001) compared to control participants (Table 4). These associations were observed in all KD patients, irrespective of coronary artery abnormalities. There was limited evidence of a similar association with Australian KD patients having larger retinal arterioles compared to control participants (155.90 ± 12.15 versus 151.99 ± 15.78 μm, p = 0.14). Australian KD patients with coronary artery abnormalities had marginally larger total fractal dimension (1.51 ± 0.04 vs. 1.49 ± 0.05, p = 0.02) compared to control participants, attributable to a larger arteriole fractal dimension.

The associations and magnitude of difference found in the univariate comparison remained similar after adjusting for age, sex, mean arterial blood pressure, and body mass index (Table 5). The mean difference in vessel tortuosity in the Singaporean cohort remained unchanged when the size of the retinal venule or arteriole was added to the multi-regression model. The adjusted mean differences in retinal microvascular parameters between Singaporean KD and control participants remained similar when the 18 Malay and 18 Indians were removed from the analysis, leaving 126 Chinese participants. Similarly, the adjusted mean difference in retinal microvascular parameters between Australian KD and control participants remain unchanged when data from 84 Caucasians were analysed, having removed 19 Asian, 5 non-Caucasian or Asian, and 8 mixed ethnicity participants (data not shown).

There was no difference in hsCRP between Australian KD patients and control participants (0.60 mg/L, 95% CI 0.41 to 0.88 versus 0.49 mg/L, 95%CI 0.36 to 0.68, p = 0.4) and no evidence of an association between hsCRP and size of retinal venules (p = 0.18) (Supplementary Figure 3).

## Discussion

This is the first study to describe changes in the retinal vasculature following KD. We found larger retinal venules in Singaporean KD patients, with an incremental increase in those patients with coronary artery abnormalities. There was limited evidence for analogous changes in Australian KD patients. The differences in retinal venular calibre were unchanged after adjustment for age, sex, mean arterial pressure, and body mass index. Additional retinal microvascular changes were cohort-specific, with larger retinal arterioles, reduced arteriolar and venular tortuosity in Singaporean KD patients, and larger fractal dimensions in Australian KD patients compared to respective control participants. Our study provides the first evidence that following KD, patients have observable differences in their retinal microvasculature.

Retinal microvascular calibre has well-established reproducibility and associations with cardiovascular risk factors and outcomes^8^,^10^,^15^. Larger retinal venules have been associated with endothelial dysfunction, oxidative stress and various biomarkers of chronic inflammation, which are key players in the pathophysiology of vascular injury^11^,^12^. Increased biomarkers of oxidative stress and inflammation have been reported following KD especially in patients with coronary artery abnormalities, suggesting a potential role for retinal venular changes as an additional marker of future cardiovascular risk in KD^25^,^26^.

There was a lack of consistency in the observed differences in the retinal venules between cases and controls in the Australian and Singaporean cohorts. Ethnic differences in KD susceptibility are well-recognised^2^. Inclusion of both Caucasian and Asian cases and controls afforded the opportunity to examine putative ethnic differences in microvascular outcomes. Significantly larger retinal venules were detected in Singaporean patients, despite having over three-fold fewer patients with coronary artery abnormalities compared to the Australian cohort. The limited published data suggest that vascular changes post KD may be more adverse in those of Asian ethnicity compared to Caucasians, although direct comparison between studies is difficult. Carotid artery intima-media thickness (an intermediate phenotype of cardiovascular risk) is increased in Asians following KD despite small samples sizes, whereas studies of Caucasians with larger samples have generally reported no differences^27^. The smaller Australian sample size may not have power to detect small differences in retinal venules, if the changes in Caucasian KD patients are less marked. Given the heterogeneity of retinal microvascular parameters, it is also possible that these differences may be due to chance, or that they preceded the KD illness.

Singaporean KD patients had larger retinal arterioles compared to control participants, with an incremental increase in those with coronary artery abnormalities. Larger retinal arteriole may reflect chronic inflammation, as larger arteriolar calibre has been reported with higher leucocyte counts and higher erythrocyte sedimentation rate^9^. Further studies that combine retinal assessment with biomarker analysis are warranted. Of note, we observed lower blood pressures in Singaporean KD patients compared to control participants, which has been previously reported following KD, although the cause is uncertain and may be spurious^28^. To account for this difference, the mean arterial blood pressure was included as a co-variate in the multi-variable linear regression model investigating differences in the retinal microvasculature.

The mechanisms underlying differences in geometric parameters (lesser microvascular tortuosity and increased fractal dimensions) and their clinical significance are less clear. There are fewer data on these parameters with considerable variation in methodologies. Cardiovascular risk and outcomes have been associated with either ends of measurement (e.g. highest and lowest quartiles of fractal dimension, increased and decreased arterial tortuosity) suggesting the possibility of an optimal range in these geometric parameters^17^,^18^.

Adult epidemiological studies, in general, show narrower retinal vascular calibre with age^29^. There is limited evidence of an effect of age on retinal venular calibre in our study. It is therefore unlikely that the increase in retinal venular calibre in Singaporean KD patients is due to a marginally older control group (mean difference 1.4 years, 95% CI 0.5 to 2.3).

Data on hsCRP was not available for the Singaporean cohort in whom larger differences in retinal venules between KD patients and control participants were observed. For the Australian KD cohort there were no difference in hsCRP in the post-acute phase, nor evidence of an association between hsCRP and retinal venular diameter. High sensitivity CRP is an acute phase reactant in children and levels return to within the normal range after an acute illness; it may therefore be a sub-optimal marker of chronic inflammation in children^30^.

The strengths of our study include the use of quantitative detailed measurements of retinal microvascular parameters by a standardised computer-assisted program in two ethnic cohorts.

Limitations include differences in the clinical characteristics of KD patients and differences in retinal parameters between control groups from the two countries; ethnic differences in retinal parameters have been described before^7^. To minimize the impact of this, data from each cohort was statistically analysed and presented separately, adjusting for magnification differences in the retinal cameras. All retinal photographs were analysed by same graders using a standardised method. We did not collect data on refractive error for all KD patients and Australian control participants. Refractive error may affect retinal microvascular parameters but its effect on the observed differences in the retinal microvascular calibre is likely to be insignificant^31^. Furthermore, there are limitations with self-reporting of ethnicity and we cannot exclude a more diverse ethnic mix than was reported. This study has inherent limitations due to its retrospective cross-sectional design; causal relationships and long term changes of these retinal parameters cannot be examined. The long-term implications of the differences in retinal microvascular parameters are unknown.

## Conclusion

Singaporean KD patients have a modest increase in the calibre of their retinal venules. There was limited evidence of analogous differences in Australian KD patients. In adults, larger retinal venules are associated with inflammation, cardiovascular risk factors and incident cardiovascular events^11^,^12^,^16^,^29^. Other differences in retinal vascular parameters were cohort-specific and their clinical significance are unknown. Prospective longitudinal studies in KD patients are needed to confirm these changes in retinal microvascular parameters over time, especially in light of the inconsistencies between the two cohorts, and the association with later cardiovascular risk.

## Additional Information

**How to cite this article:** Chen, K. Y. H. *et al*. Evidence of Microvascular Changes in the Retina following Kawasaki disease. *Sci. Rep.* **7**, 40513; doi: 10.1038/srep40513 (2017).

**Publisher's note:** Springer Nature remains neutral with regard to jurisdictional claims in published maps and institutional affiliations.

## Supplementary Material

Supplementary Information

## Figures and Tables

**Table 1:** Method of measurement for individual retinal microvascular parameters.

Retinal microvascular parameter	Method of measurement
Calibre	The width of retinal arterioles and venules in a pre-defined zone are measured based on the revised Knudtson-Parr-Hubbard formula. Retinal arteriolar and venular calibres are summarised as the central retinal artery equivalent (CRAE) and central retinal vein equivalent (CRVE), respectively. The ratio of both variables is the arterio-venous ratio (AVR).
Branching Angle	The first angle subtended between two daughter vessels at each bifurcation. The estimates are summarised as retinal arteriolar branching angle and retinal venular branching angle, representing the average branching angle of arterioles and venules of the eye, respectively.
Fractal dimension	Skeletonized line tracing using the box-counting method is used to calculate the fractal dimension, which summarized the whole branching pattern of the retinal microvascular tree. Larger values indicate a more complex branching pattern.
Tortuosity	The relative length variation between the curvatures of the vessel versus the shortest distance of the vessel path. The estimates are summarised as retinal arteriolar tortuosity and retinal venular tortuosity, representing the average tortuosity of the arterioles and venules in the eye, respectively. Smaller tortuosity values indicate straighter vessels.

**Table 2:** Demographics and cardiovascular risk factors.

	Australian KD (n = 57)	Australian Controls (n = 59)	Australian KD vs controls p value	Singaporean KD (n = 78)	Singaporean controls (n = 78)	Singaporean KD vs controls p value
Age (years)	15.5 ± 5.6	15.2 ± 6.3	0.8	10.2 ± 3.8	11.6 ± 1.2	0.002
Male (n (%))	33 (57.9)	26 (44.1)	0.14	48 (62.0)	50 (64.0)	0.9
European-Caucasians (n (%))	42 (73.7)	42 (71.2)	0.7	0	0	
Asian (n (%)) (South east Asians, Indians)	10 (17.5)	9 (15.3)		78 (100)	78 (100)	
Others (n (%)) (Africans, Latin Americans, Polynesians)	1 (1.8)	4 (6.8)		0	0	
Mixed (n (%)) parents from 2 different above groups	4 (7.0)	4 (6.8)		0	0	
Systolic blood pressure (mmHg)	115.9 ± 9.9	116.7 ± 9.9	0.7	106.8 ± 12.2	112.1 ± 14.8	0.02
Diastolic blood pressure (mmHg)	66.1 ± 6.6	67.4 ± 8.0	0.4	60.8 ± 6.8	65.3 ± 9.7	0.001
Body mass index	20.6 ± 4.4	20.6 ± 3.5	0.9	18.8 ± 3.5	19.7 ± 3.8	0.13
Current smokers (n (%))	3 (5.2)	1 (1.7)	1	0	0	
*Geometric mean hsCRP (mg/L)	0.60 (95% CI 0.41, 0.88)	0.49 (95%CI 0.36, 0.68)	0.4	N/A		

Mean ± SD unless otherwise specified, KD = Kawasaki disease, N/A = not available, hsCRP = high sensitivity C reactive protein. *Log transformed hsCRP values have been retransformed back to mg/L using the exponential function to give geometric means and 95% confidence intervals.

**Table 3:** Clinical characteristics of Kawasaki disease patients.

	Australian KD (n = 57)	Singaporean KD (n = 78)	p value
Age at diagnosis (years)	3.6 ± 3.2	2.9 ± 2.4	0.21
Time since acute illness (years)	11.8 ± 5.9	6.9 ± 4.0	<0.001
N (%) treated with IVIG	51 (89.5)	48 (61.5)	0.21
Unknown if treated with IVIG	1 (1.8)	28 (35.9)	
N (%) with always normal coronary arteries	25 (43.9)	66 (84.6)	<0.001
N (%) with CA abnormalities <8 mm	23 (40.4)	11 (14.1)	
Persistent CA abnormalities < 8 mm	6 (10.5)	6 (7.7)	
Regressed CA abnormalities < 8 mm	17 (27.8)	5 (6.4)	
N (%) with CA abnormalities > or equal to 8 mm	8 (14.0)	0	<0.001
Persistent CA abnormalities > or equal to 8 mm	8 (14.0)		
Regressed CA abnormalities > or equal to 8 mm	0		
Coronary artery diameter unknown	1 (1.8)	1 (1.3)	

Mean ± SD unless otherwise specified, KD = Kawasaki disease, CA = coronary artery.

**Table 4:** Univariate comparison of retinal microvascular parameters.

	Controls (n = 78)	All KD (n = 78)	All KD vs controls p value	KD always normal CA (n = 66)	KD always normal CA vs control p value	KD ^+^CA abnormalities (n = 11)	KD CA abnormalities vs control p value
**Australian**							
CRAE (μm)	151.99 ± 15.78	155.90 ± 12.15	0.14	156.72 ± 9.99	0.17	155.27 ± 13.72	0.33
CRVE (μm)	214.38 ± 22.69	218.83 ± 14.54	0.21	215.81 ± 13.20	0.77	221.20 ± 15.30	0.13
AVR	0.71 ± 0.04	0.71 ± 0.05	0.77	0.73 ± 0.05	0.13	0.70 ± 0.05	0.41
Arteriolar branching angle (deg)	83.71 ± 9.14	84.99 ± 9.45	0.46	84.83 ± 9.91	0.62	85.11 ± 9.26	0.49
Venular branching angle (deg)	78.80 ± 8.16	80.71 ± 9.12	0.24	79.71 ± 10.15	0.67	81.50 ± 8.31	0.14
Total fractal dimension	1.49 ± 0.05	1.50 ± 0.05	0.13	1.49 ± 0.06	0.88	1.51 ± 0.04	0.02
Fractal dimension arteriole	1.28 ± 0.05	1.30 ± 0.05	0.07	1.29 ± 0.06	0.43	1.31 ± 0.04	0.03
Fractal dimension venule	1.25 ± 0.04	1.26 ± 0.06	0.42	1.24 ± 0.06	0.58	1.27 ± 0.05	0.07
*Geometric mean arteriolar tortuosity (x10^−5^)	8.36 (95% CI 7.71, 9.05)	8.36 (95% CI 7.87, 8.70)	0.89	8.11 (95% CI 7.41, 8.78)	0.57	8.52 (95% CI 7.87, 9.23)	0.78
*Geometric mean venular tortuosity (x10^−5^)	7.95 (95% CI 7.49, 8.52)	7.49 (95% CI 7.19, 7.71)	0.09	7.49 (95% CI 6.98, 7.95)	0.22	7.49 (95% CI 7.19, 7.87)	0.19
**Singaporean**							
CRAE (μm)	140.15 ± 9.75	149.43 ± 13.72	<0.001	148.78 ± 13.30	<0.001	155.14 ± 14.91	<0.001
CRVE (μm)	205.31 ± 13.65	216.25 ± 14.41	<0.001	215.01 ± 13.60	<0.001	225.63 ± 15.56	<0.001
AVR	0.68 ± 0.04	0.69 ± 0.05	0.3	0.69 ± 0.05	0.3	0.69 ± 0.04	0.8
Arteriolar branching angle (deg)	82.41 ± 8.51	81.35 ± 9.61	0.5	80.64 ± 8.24	0.2	85.37 ± 15.77	0.3
Venular branching angle (deg)	79.59 ± 7.70	80.55 ± 7.60	0.4	81.01 ± 7.62	0.3	78.09 ± 7.66	0.5
Total fractal dimension	1.51 ± 0.03	1.50 ± 0.04	0.4	1.50 ± 0.04	0.4	1.50 ± 0.03	0.5
Fractal dimension arteriole	1.30 ± 0.04	1.29 ± 0.05	0.3	1.30 ± 0.05	0.2	1.30 ± 0.04	1.0
Fractal dimension venule	1.27 ± 0.04	1.27 ± 0.04	0.8	1.27 ± 0.05	0.9	1.26 ± 0.03	0.2
*Geometric mean arteriolar tortuosity (x10^−5^)	8.70 (95% CI 8.36, 9.14)	7.34 (95% CI 7.05, 7.64)	<0.001	7.34 (95% CI 6.98, 7.64)	<0.001	7.41 (95% CI 6.57, 8.44)	0.01
*Geometric mean venular tortuosity (x10^−5^)	9.42 (95% CI 9.14, 9.71)	7.34 (95% CI 7.12, 7.64)	<0.001	7.34 (95% CI 7.12, 7.64)	<0.001	7.49 (95% CI 6.77, 8.27)	<0.001

Mean ± SD unless otherwise specified, KD = Kawasaki disease, CRAE = central retinal artery equivalent, CRVE = central retinal vein equivalent, AVR = arterio-venous ratio, CA = coronary artery. ^+^“CA abnormalities” includes patients with regressed and persistent coronary artery abnormalities. *Log transformed tortuosity values have been retransformed using the exponential function to give geometric means and 95% confidence intervals.

**Table 5:** Adjusted^#^ mean difference of retinal microvascular parameters.

	All KD (n = 78)	All KD vs control p value	KD always normal CA (n = 66)	KD always normal CA vs control p value	KD^+^ CA abnormalities (n = 11)	KD CA abnormalities vs control p value
**Australian**						
CRAE (μm)	4.29 (−1.04, 9.63)	0.11	5.05 (−1.82, 11.94)	0.15	3.68 (−2.71, 10.07)	0.26
CRVE (μm)	4.89 (−2.35, 12.14)	0.18	1.86 (−7.45, 11.17)	0.69	7.34 (−1.30, 15.99)	0.10
AVR	0.003 (−0.01, 0.02)	0.73	0.02 (−0.006, 0.04)	0.13	−0.008 (−0.03, 0.01)	0.43
Arteriolar branching angle (deg)	1.50 (−2.03, 5.02)	0.40	1.01 (−3.54, 5.56)	0.66	1.88 (−2.34, 6.11)	0.38
Venular branching angle (deg)	1.55 (−1.63, 4.73)	0.34	0.62 (−3.48, 4.72)	0.77	2.30 (−1.51, 6.10)	0.23
Total Fractal dimension	0.01 (−0.003, 0.03)	0.11	0.002 (−0.02, 0.03)	0.84	0.02 (0.003, 0.05)	0.03
Fractal dimension arteriole	0.02 (−0.0002, 0.04)	0.05	0.01 (−0.01, 0.04)	0.32	0.02 (0.001, 0.05)	0.04
Fractal dimension venule	0.008 (−0.01, 0.03)	0.43	−0.007 (−0.03, 0.02)	0.54	0.02 (−0.002, 0.04)	0.08
*Geometric mean arteriolar tortuosity	0.98 (0.89, 1.08)	0.73	0.94 (0.85, 1.05)	0.29	1.02 (0.91, 1.14)	0.76
*Geometric mean venular tortuosity	0.95 (0.88, 1.02)	0.14	0.94 (0.86, 1.03)	0.20	0.95 (0.88, 1.02)	0.18
**Singaporean**						
CRAE (μm)	8.33 (4.23, 12.43)	<0.001	7.55 (3.35, 11.74)	0.001	14.36 (5.85, 22.88)	0.001
CRVE (μm)	9.67 (4.87, 14.51)	<0.001	8.49 (3.60, 13.39)	0.001	18.92 (8.98, 28.86)	<0.001
AVR	0.007 (−0.009, 0.02)	0.39	0.006 (−0.01, 0.02)	0.39	0.005 (−0.03, 0.04)	0.77
Arteriolar branching angle (deg)	−1.60 (−4.71, 1.50)	0.31	−2.24 (−5.41, 0.94)	0.17	3.30 (−3.14, 9.74)	0.31
Venular branching angle (deg)	0.96 (−1.69, 3.61)	0.48	1.30 (−1.43, 4.02)	0.35	−1.64 (−7.18, 3.89)	0.56
Total fractal dimension	−0.01 (−0.02, 0.007)	0.42	−0.01 (−0.02, 0.007)	0.42	−0.004 (−0.03, 0.02)	0.76
Fractal dimension arteriole	−0.01 (−0.03, 0.009)	0.43	−0.01 (−0.03, 0.008)	0.35	0.004 (−0.03, 0.03)	0.78
Fractal dimension venule	−0.002 (−0.02, 0.01)	0.78	−0.006 (−0.01, 0.01)	0.93	−0.01 (−0.04, 0.02)	0.39
*Geometric mean ratio arteriolar tortuosity	0.83 (0.78, 0.89)	<0.001	0.83 (0.78, 0.89)	<0.001	0.83 (0.72, 0.94)	0.005
*Geometric mean ratio venular tortuosity	0.79 (0.75, 0.83)	<0.001	0.79 (0.75, 0.83)	<0.001	0.81 (0.73, 0.90)	<0.001

Data presented as mean difference (95% CI of difference) ^#^Multivariable regression model adjusted for age, sex, mean arterial blood pressure, and body mass index. KD = Kawasaki disease, CRAE = central retinal artery equivalent, CRVE = central retinal vein equivalent, AVR = arterio-venous ratio, CA = coronary artery. ^+^“CA abnormalities” includes patients with regressed and persistent coronary artery abnormalities. *Log transformed tortuosity values have been retransformed using the exponential function to give geometric means and 95% confidence intervals.

## References

[b1] Kawasaki T. Acute febrile mucocutaneous syndrome with lymphoid involvement with specific desquamation of the fingers and toes in children. Jpn J Allergy 16, 178–222 (1967). 6062087

[b2] Uehara R. & Belay E. D. Epidemiology of Kawasaki disease in Asia, Europe, and the United States. J Epidemiol 22, 79–85 (2012). 22307434 10.2188/jea.JE20110131 PMC3798585

[b3] Chen K. Y. . Kawasaki Disease and Cardiovascular Risk: A Comprehensive Review of Subclinical Vascular Changes in the Longer Term. Acta Paediatr 105, 752–761 (2016). 26880292 10.1111/apa.13367

[b4] Hoshino S., Tsuda E. & Yamada O. Characteristics and Fate of Systemic Artery Aneurysm after Kawasaki Disease. The Journal of Pediatrics 167, 108–112 (2015). 25981909 10.1016/j.jpeds.2015.04.036

[b5] Gishti O. . Retinal microvasculature and cardiovascular health in childhood. Pediatrics 135, 678–685 (2015). 25755243 10.1542/peds.2014-3341

[b6] Strain W. D., Adingupu D. D. & Shore A. C. Microcirculation on a large scale: techniques, tactics and relevance of studying the microcirculation in larger population samples. Microcirculation 19, 37–46 (2012). 21972935 10.1111/j.1549-8719.2011.00140.x

[b7] Cheung N. . Distribution and Associations of Retinal Vascular Caliber with Ethnicity, Gender, and Birth Parameters in Young Children. Invest Ophthalmol Vis Sci 48, 1018–1024 (2007). 17325141 10.1167/iovs.06-0978

[b8] Couper D. J. . Reliability of retinal photography in the assessment of retinal microvascular characteristics: the Atherosclerosis Risk in Communities Study. Am J Ophthalmol 133, 78–88 (2002). 11755842 10.1016/s0002-9394(01)01315-0

[b9] Ikram M. K. . Are Retinal Arteriolar or Venular Diameters Associated with Markers for Cardiovascular Disorders? The Rotterdam Study. Invest Ophthalmol Vis Sci 45, 2129–2134 (2004). 15223786 10.1167/iovs.03-1390

[b10] Wong T. Y. . Retinal microvascular abnormalities and incident stroke: the Atherosclerosis Risk in Communities Study. The Lancet 358, 1134–1140 (2001). 10.1016/S0140-6736(01)06253-5 11597667

[b11] Daien V. . Retinal Vascular Caliber Is Associated with Cardiovascular Biomarkers of Oxidative Stress and Inflammation: The POLA Study. PLoS One 8, e71089 (2013). 23923054 10.1371/journal.pone.0071089 PMC3724806

[b12] Klein R., Klein B. K., Knudtson M. D., Wong T. Y. & Tsai M. Y. Are inflammatory factors related to retinal vessel caliber?: The Beaver Dam Eye Study. Arch Ophthalmol 124, 87–94 (2006). 16401789 10.1001/archopht.124.1.87

[b13] Cheung C. Y.-l. . C-reactive protein and retinal microvascular caliber in a multiethnic asian population. Am J Epidemiol 171, 206–213 (2010). 20007993 10.1093/aje/kwp357

[b14] Hanssen H. . Retinal vessel diameter, obesity and metabolic risk factors in school children (JuvenTUM 3). Atherosclerosis 221, 242–248 (2012). 22244041 10.1016/j.atherosclerosis.2011.12.029

[b15] Wong T. Y. . Quantitative retinal venular caliber and risk of cardiovascular disease in older persons: the cardiovascular health study. Arch Intern Med 166, 2388–2394 (2006). 17130394 10.1001/archinte.166.21.2388

[b16] Cheung C. Y. . Retinal Microvascular Changes and Risk of Stroke: The Singapore Malay Eye Study. Stroke 44, 2402–2408 (2013). 23868266 10.1161/STROKEAHA.113.001738

[b17] Liew G. . Fractal analysis of retinal microvasculature and coronary heart disease mortality. Eur Heart J 32, 422–429 (2011). 21138936 10.1093/eurheartj/ehq431

[b18] Witt N. . Abnormalities of retinal microvascular structure and risk of mortality from ischemic heart disease and stroke. Hypertension 47, 975–981 (2006). 16585415 10.1161/01.HYP.0000216717.72048.6c

[b19] Tapp R. J. . Impact of blood pressure on retinal microvasculature architecture across the lifespan: the Young Finns Study. Microcirculation 22, 146–155 (2015). 25559612 10.1111/micc.12187

[b20] Newburger J. W. . Diagnosis, treatment, and long-term management of Kawasaki disease: A statement for health professionals from the Committee on Rheumatic Fever, Endocarditis and Kawasaki Disease, Council on Cardiovascular Disease in the Young, American Heart Association. Circulation 110, 2747–2771 (2004). 15505111 10.1161/01.CIR.0000145143.19711.78

[b21] JCS Joint Working Group. Guidelines for Diagnosis and Management of Cardiovascular Sequelae in Kawasaki Disease (JCS 2013). *Circ J* **78**, 2521–2562 (2014). 10.1253/circj.cj-66-0096 25241888

[b22] Kurniawan E. D. . The Relationship between Changes in Body Mass Index and Retinal Vascular Caliber in Children. The Journal of Pediatrics 165, 1166–1171.e1161 (2014). 25262303 10.1016/j.jpeds.2014.08.033

[b23] Cheung C. Y.-l. . A New Method to Measure Peripheral Retinal Vascular Caliber over an Extended Area. Microcirculation 17, 495–503 (2010). 21040115 10.1111/j.1549-8719.2010.00048.x

[b24] Sasongko M. B. . Correlation and reproducibility of retinal vascular geometric measurements for stereoscopic retinal images of the same eyes. Ophthalmic Epidemiol 19, 322–327 (2012). 22978533 10.3109/09286586.2012.702258

[b25] Cheung Y. F., Ho M. H. K., Tam S. C. F. & Yung T. C. Increased high sensitivity C reactive protein concentrations and increased arterial stiffness in children with a history of Kawasaki disease. Heart 90, 1281–1285 (2004). 15486121 10.1136/hrt.2003.018507 PMC1768534

[b26] Mitani Y. . Elevated levels of high-sensitivity C-reactive protein and serum amyloid-A late after Kawasaki disease: association between inflammation and late coronary sequelae in Kawasaki disease. Circulation 111, 38–43 (2005). 15611368 10.1161/01.CIR.0000151311.38708.29

[b27] Dietz S. M., Tacke C. E., Hutten B. A. & Kuijpers T. W. Peripheral Endothelial (Dys) Function, Arterial Stiffness and Carotid Intima-Media Thickness in Patients after Kawasaki Disease: A Systematic Review and Meta-Analyses. PLoS One 10, e0130913 (2015). 26161871 10.1371/journal.pone.0130913 PMC4498761

[b28] Vaujois L. . The biophysical properties of the aorta are altered following Kawasaki disease. J Am Soc Echocardiogr 26, 1388–1396 (2013). 24094561 10.1016/j.echo.2013.08.022

[b29] Wong T. Y. . Retinal Vascular Caliber, Cardiovascular Risk Factors, and Inflammation: The Multi-Ethnic Study of Atherosclerosis (MESA). Invest Ophthalmol Vis Sci 47, 2341–2350 (2006). 16723443 10.1167/iovs.05-1539 PMC2258139

[b30] Urbach J., Shapira I., Branski D. & Berliner S. Acute phase response in the diagnosis of bacterial infections in children. Pediatr Infect Dis J 23, 159–160 (2004). 14872184 10.1097/01.inf.0000115735.78960.a4

[b31] Wong T. Y., Wang J. J., Rochtchina E., Klein R. & Mitchell P. Does refractive error influence the association of blood pressure and retinal vessel diameters? The blue mountains eye study. Am J Ophthalmol 137, 1050–1055 (2004). 15183789 10.1016/j.ajo.2004.01.035

