# Supplementary material for: Evidence of Microvascular Changes in the Retina following Kawasaki Disease
Source: Sci Rep. 2017 Jan 17;7:40513. doi: 10.1038/srep40513 (PMC5240332; doi:10.1038/srep40513)
Supplement: Supplementary Information [file srep40513-s1.pdf]

ONLINE SUPPLEMENTAL MATERIAL

Title: Evidence of Microvascular Changes in the Retina following Kawasaki disease

Authors: Katherine YH Chen MBBS[1, 2, 3] David P Burgner PhD[1, 2, 4*] Tien Y Wong PhD[5,6] Seang Mei Saw PhD[5,6] Swee Chye Quek MD[7,8] Audrey YC Pang FRCOphth [9] Seo Wei Leo FRCSEd(Ophth) [10] Inez B Wong FRCSEd(Ophth)[11] Diana Zannino MSc [1] Nigel Curtis PhD[1,2, 3] Michael Cheung MD[1, 2, 12] Carol Y Cheung PhD[5,13#] Terence CW Lim MRCPCH [7,8#]

Supplementary 1. Differences in microvascular parameters between Australian and Singaporean cohorts

	Australian controls (n= 59)	Singaporean controls (n= 78)	Australian vs Singaporean controls p value	Australian KD (n= 57)	Singaporean KD (n= 78)	Australian vs Singaporean KD p value
**CRAE (µm)**	151.99 ± 15.78	140.15 ± 9.75	<0.001	155.90 ± 12.15	149.43 ± 13.72	0.005
**CRVE (µm)**	214.38 ± 22.69	205.31 ± 13.65	0.004	218.83 ± 14.54	216.25 ± 14.41	0.31
**AVR**	0.71 ± 0.04	0.68 ± 0.04	<0.001	0.71 ± 0.05	0.69 ± 0.05	0.007
**Arteriolar branching angle (deg)**	83.71 ± 9.14	82.41 ± 8.51	0.40	84.99 ± 9.45	81.35 ± 9.61	0.03
**Venular branching angle (deg)**	78.80 ± 8.16	79.59 ± 7.70	0.56	80.71 ± 9.12	80.55 ± 7.60	0.90
**Total Fractal dimension**	1.49 ± 0.05	1.51 ± 0.03	0.002	1.50 ± 0.05	1.50 ± 0.04	0.76
**Fractal dimension arteriole**	1.28 ± 0.05	1.30 ± 0.04	0.03	1.30 ± 0.05	1.29 ± 0.05	0.34
**Fractal dimension venule**	1.25 ± 0.04	1.27 ± 0.04	0.002	1.26 ± 0.06	1.27 ± 0.04	0.13
***Geometric mean arteriolar tortuosity (x10^{-5})**	8.36 (95% CI 7.71, 9.05)	8.70 (95% CI 8.36, 9.14)	0.33	8.36 (95% CI 7.87, 8.78)	7.34 (95% CI 7.05, 7.64)	<0.001

***Geometric mean venular tortuosity (x10^{-5})**	7.95 (95% CI 7.49, 8.52)	9.42 (95% CI 9.14, 9.71)	<0.001	7.49 (95% CI 7.19, 7.71)	7.34 (95% CI 7.12, 7.64)	0.50

Mean ± SD unless otherwise specified, KD= Kawasaki disease, CRAE= central retinal artery equivalent, CRVE= central retinal vein equivalent, AVR = arterio-venous ratio, *Log transformed tortuosity values have been retransformed using the exponential function to give geometric means and 95% confidence intervals.

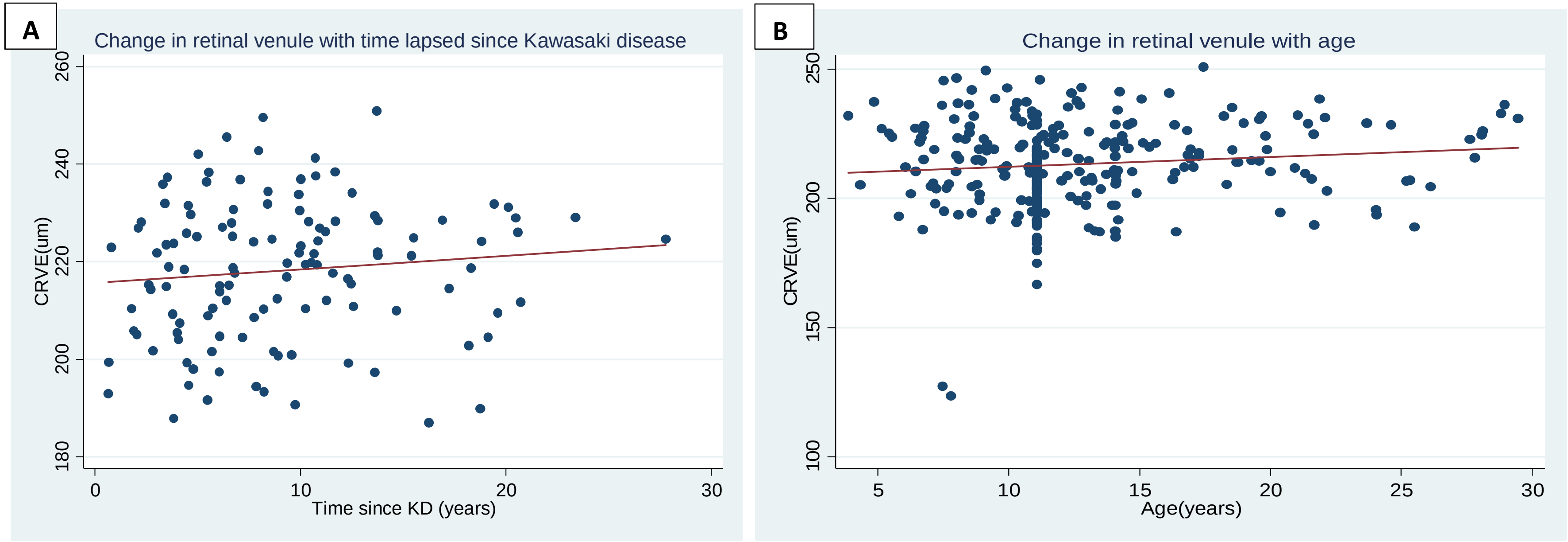

Supplementary 2. A. Scatter plot of the association between size of retinal venules and time since Kawasaki disease in 57 Australian KD patients and 78 Singaporean KD patients. B. Scatter plot of the association between size of retinal venules and age in all 135 KD patients and all 137 controls. (KD= Kawasaki disease, CRVE= central retinal vein equivalent)

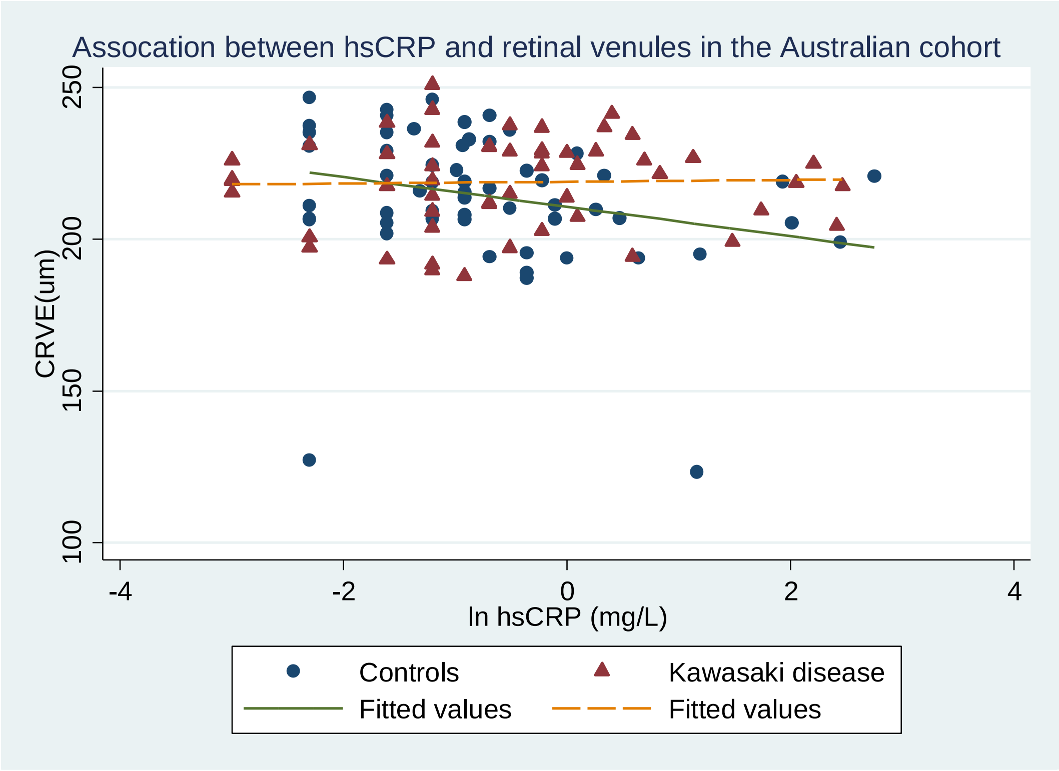

Supplementary 3. Scatter plot of the association between high sensitivity C-reactive protein and retinal venules in 57 Kawasaki disease patients and 59 controls. (hsCRP= high sensitivity C-reactive protein, CRVE= central retinal vein equivalent)
